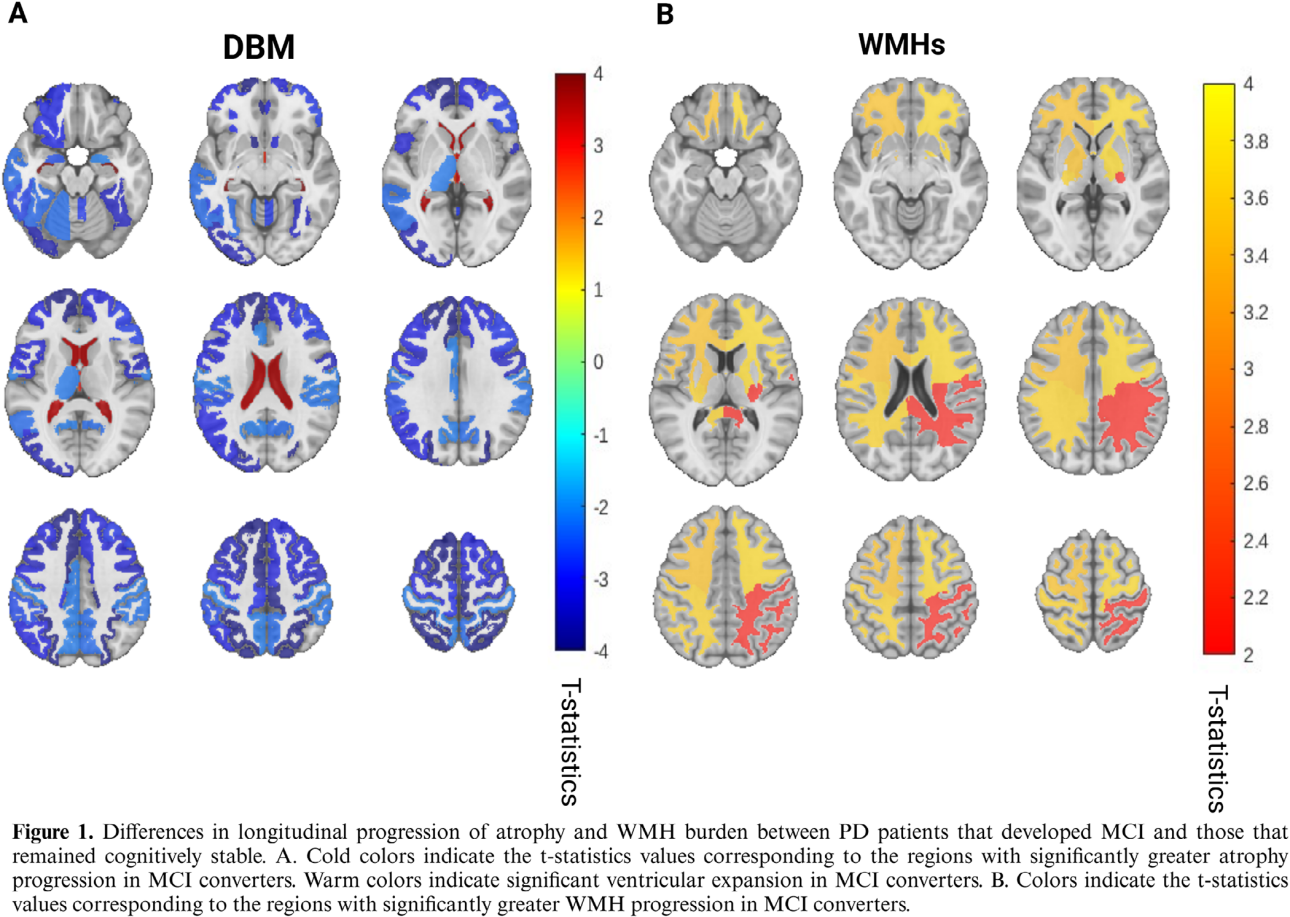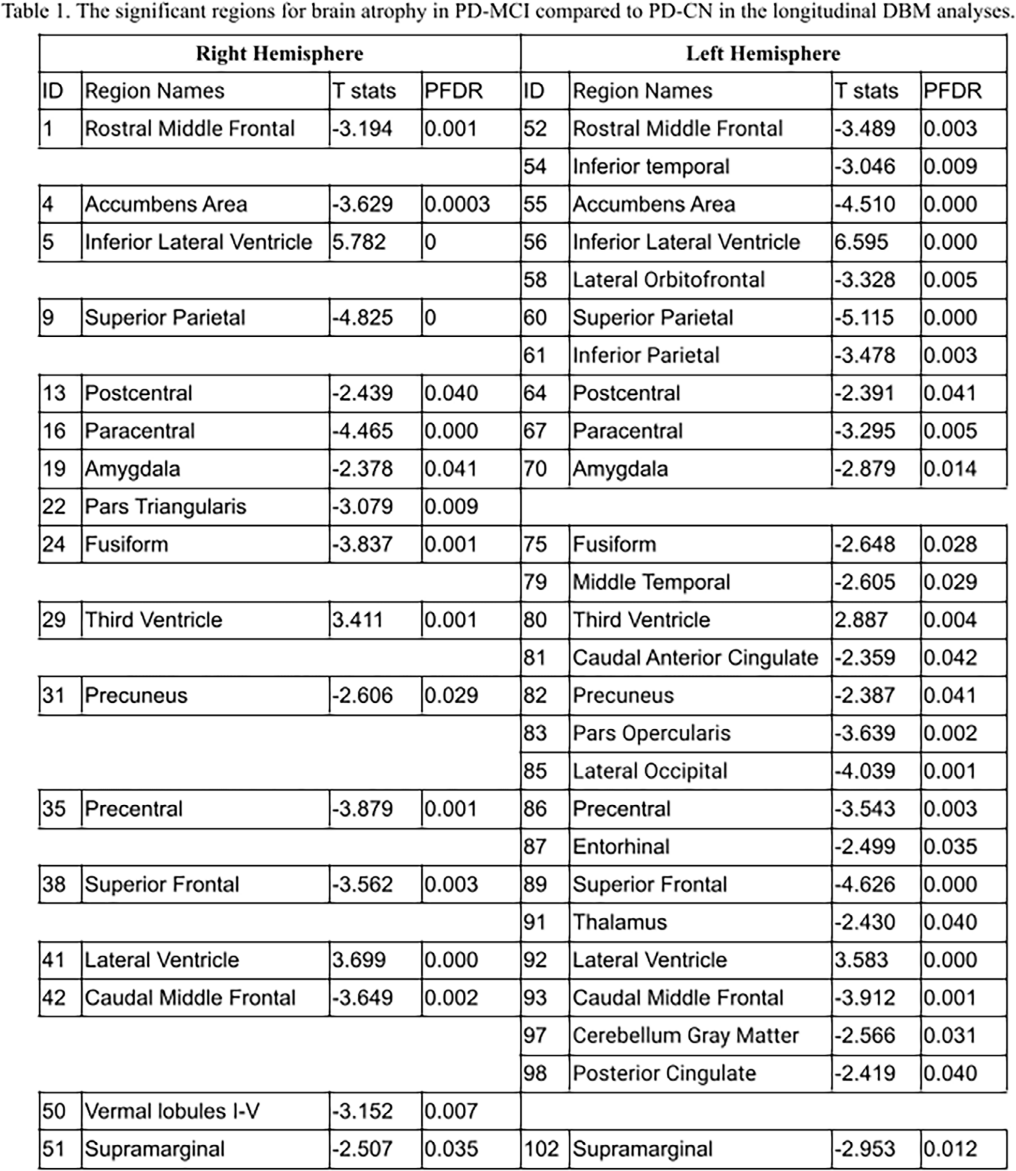# Gray and white matter pathology progression in Parkinson's disease patients that develop mild cognitive impairment

**DOI:** 10.1002/alz70856_106745

**Published:** 2026-01-10

**Authors:** Roqaie Moqadam, Houman Azizi, Yashar Zeighami, Mahsa Dadar

**Affiliations:** ^1^ University of Montreal, Montréal, QC, Canada; ^2^ Douglas Mental Health University Institute, Montréal, QC, Canada; ^3^ Institut Universitaire de Gériatrie de Montréal, Montréal, QC, Canada; ^4^ McGill University, Montreal, QC, Canada; ^5^ Douglas Mental Health University Institute, Montreal, QC, Canada; ^6^ The Neuro (Montreal Neurological Institute), Montreal, QC, Canada; ^7^ Department of Psychiatry, McGill University, Montréal, QC, Canada

## Abstract

**Background:**

Cognitive impairment is a common non‐motor symptom in Parkinson's disease (PD) that can potentially occur at any disease stage (Aarsland et al., 2021). Compared to cognitively normal patients, PD patients that develop mild cognitive impairment (MCI) show greater levels of atrophy (Mak et al., 2015). Greater baseline white matter hyperintensity (WMH) burden is also linked to more severe future cognitive deficits in PD patients (Dadar et al., 2018). Here, we assess the longitudinal changes in gray matter (GM) atrophy and WMH burden in PD patients that develop MCI compared to those that remain cognitively normal.

**Methods:**

Imaging and clinical data were obtained from the Parkinson's Progression Markers Initiative (PPMI) study (Marek et al., 2018). Deformation‐based morphometry (DBM) maps and WMH segmentations were extracted using T1w images in 312 PD (559 timepoints) using an in‐house pipeline (Lajoie et al. 2025) and BISON (Dadar et al., 2021), respectively. A series of linear mixed‐effects models were used to assess the differences in the longitudinal trajectories of the brain measures (DBM and WMHs) between the patients that developed MCI compared to those that remained stable using an interaction term between conversion_status and time from baseline visit as the variable of interest. The models included MoCA score at baseline, sex, and age at the baseline as covariates. False Discovery Rate (FDR) method was applied for multiple comparisons correction (Benjamini and Hochberg, 1995).

**Results:**

All included PD patients were cognitively normal at baseline and had 6 years of follow‐up assessments available for cognitive status. During these 6 years, 47 patients developed MCI, while 265 remained cognitively normal. Figures 1A‐B show the t‐statistic maps of the regions that showed significantly greater rates of longitudinal atrophy, ventricular expansion, and WMH progression in patients that developed MCI compared against those that remained cognitively healthy following FDR correction. The observed differences for both atrophy and WMHs were the strongest in bilateral frontal regions as well as the accumbens areas (Table 1).

**Conclusions:**

Our results suggest that PD patients that develop MCI experience greater levels of pathology in both gray and white matter brain regions in the frontal lobes.